# “No Way Out Except From External Intervention”: First-Hand Accounts of Autistic Inertia

**DOI:** 10.3389/fpsyg.2021.631596

**Published:** 2021-07-13

**Authors:** Karen Leneh Buckle, Kathy Leadbitter, Ellen Poliakoff, Emma Gowen

**Affiliations:** ^1^Body, Eye and Movement Lab, Division of Neuroscience and Experimental Psychology, Faculty of Biology, Medicine and Health, Manchester Academic Health Science Centre, University of Manchester, Manchester, United Kingdom; ^2^Social Development Research Group, Division of Neuroscience and Experimental Psychology, Faculty of Biology, Medicine and Health, University of Manchester, Manchester, United Kingdom

**Keywords:** autism, movement, inertia, catatonia, qualitative, autistic adults, ASD, initiation

## Abstract

This study, called for by autistic people and led by an autistic researcher, is the first to explore ‘autistic inertia,’ a widespread and often debilitating difficulty acting on their intentions. Previous research has considered initiation only in the context of social interaction or experimental conditions. This study is unique in considering difficulty initiating tasks of any type in real life settings, and by gathering qualitative data directly from autistic people. Four face-to-face and 2 online (text) focus groups were conducted with 32 autistic adults (19 female, 8 male, and 5 other), aged 23–64 who were able to express their internal experiences in words. They articulate in detail the actions they have difficulty with, what makes it easier or harder to act, and the impact on their lives. Thematic analysis of the transcripts found four overarching themes: descriptions of inertia, scaffolding to support action, the influence of wellbeing, and the impact on day-to-day activities. Participants described difficulty starting, stopping and changing activities that was not within their conscious control. While difficulty with planning was common, a subset of participants described a profound impairment in initiating even simple actions more suggestive of a movement disorder. Prompting and compatible activity in the environment promoted action, while mental health difficulties and stress exacerbated difficulties. Inertia had pervasive effects on participants’ day-to-day activities and wellbeing. This overdue research opens the door to many areas of further investigation to better understand autistic inertia and effective support strategies.

## Introduction

Autism is a heterogeneous condition viewed as primarily a disorder of social interaction accompanied by rigid and repetitive thinking and behavior. Sensory and motor differences are mentioned only peripherally in the diagnostic criteria (American Psychiatric Association, 2013; World Health Organisation, 2020); however, sporadic but increasing research over the last 25 years has proposed that these differences may be more important in the etiology than commonly thought (Leary and Hill, 1996; Robledo et al., 2012; Donnellan et al., 2013; Torres and Donnellan, 2015; Breen and Hare, 2017; Welch et al., 2020). They have proposed rethinking some autistic traits, and associated behavioral issues such as non-compliance, as manifestations of sensory and motor differences.

An experience commonly known in the autistic community as ‘autistic inertia’ may be attributable in part to differences in motor control. ‘Inertia’ is the term for Newton’s first law of motion, which is the tendency of a body to stay in the same state of motion unless acted on by an external force. This is used metaphorically to describe difficulties both starting and stopping activities, which are commonly experienced by autistic people^1^. Inertia is described in personal blogs (Paterson, 2016; Sparrow, 2016; Buckle, 2017; Welch et al., 2020) and discussed in autistic groups and events. Inertia overlaps with the concept of ‘monotropism’ (Murray et al., 2005), or the autistic tendency to focus narrowly and deeply on topics or objects of interest, which has both positive and negative aspects. Difficulty with initiating action, specifically, is usually experienced as problematic. In response to an article on monotropism, an autistic author writes:

*To me it seems odd that inertia is often so far down the list of things that people associate with autism? […] I find [inertia] probably the single biggest problem I have that stems directly from it.* (Murray, 2017).

As evidenced by the first author’s lived experience as an autistic person who experiences severe difficulties of this nature and witnesses them in others, autistic inertia, and its effect on executing intentions, is a crucial topic to address. Although there is widespread recognition of inertia within the autistic community, and the significant effect it can have on autistic people’s daily lives, no formal research has directly investigated its nature or impact. Increased awareness and understanding of initiation impairments is particularly important as the ability to spontaneously initiate voluntary actions may underlie or influence some social and behavioral differences that are characteristic of autism.

Although there is some indication that motor difficulties are a factor, the term ‘autistic inertia’ may be an umbrella term for impairments with distinct aetiologies. For example, in her blog, Tanea Paterson, an autistic adult, describes inertia in terms of difficulty initiating movements, following instructions, and flexibly changing attentional focus (Paterson, 2016). Others describe inertia as an inability to act due to fear of unknown or undesirable outcomes. It is unclear from the existing literature whether these difficulties with initiation arise from: (i) social-emotional factors such as a primary social impairment or mental health difficulties (Hollocks et al., 2010), (ii) executive dysfunction (Ozonoff and Jensen, 1999), (iii) movement differences such as catatonia (Wing and Shah, 2006; Breen and Hare, 2017), or another mechanism as yet unidentified. These possible mechanisms are underpinned by overlapping neural circuitry, which have been found to function differently in autistic people (Abbott et al., 2018; Ozsivadjian et al., 2020).

Nearly all research on initiation in autism is intervention-based research with autistic children using the frequency of social initiation as an outcome measure. Researchers consider various mechanisms for diminished social interaction in autistic children, such as lack of social motivation (Chevallier et al., 2012; Kohls et al., 2012) or learned helplessness (Koegel and Mentis, 1985); however, none consider the possibility of an underlying deficit in the ability to initiate actions.

Mental health difficulties such as depression and anxiety occur at high rates in autistic people (Hollocks et al., 2010; Hudson et al., 2018). Avoidance due to anxiety or lack of motivation due to depression both could contribute to a lack of initiative. These issues have been linked to difficulty understanding and processing one’s emotions (alexithymia), intolerance of uncertainty (Neil et al., 2016), and cognitive inflexibility (Ozsivadjian et al., 2020). Anxiety can lead to avoidance, and depression to loss of motivation. Because of the internal, subjective nature of motivation and initiative, it is difficult to distinguish between emotional and other drivers for failure to act.

Alternatively, initiation difficulties could be due to impairments in executive function (skills involved in planning, working memory, attention, and inhibition), which have consistently been found to be impaired in autism (Ozonoff and Jensen, 1999; Bramham et al., 2009; Brandimonte et al., 2011; Demetriou et al., 2018). Monotropism is a framing of autistic attention distribution as a tendency to narrow, intense focus that contributes to autistic strengths such as expertise and enhanced detail perception (Murray et al., 2005). When viewed as a deficit, this fixed focus is known as ‘cognitive inflexibility,’ an aspect of executive dysfunction associated with anxiety and depression (Ozsivadjian et al., 2020). Differences in the cortico-striatal circuitry underlying these functions has consistently been found in autistic children and adolescents (Abbott et al., 2018; Uddin, 2021). Autistic people are also found to have impaired prospective memory, i.e., remembering to do something later. Providing a cue or initial step has been found to reduce initiation-specific deficits (Williams et al., 2014; Carmo et al., 2017). Social interaction may be particularly vulnerable to the effects of initiation impairments because it is variable and unpredictable, calling on a variety of high level flexible cognitive processes (Riggs et al., 2006).

Finally, difficulties with initiation may stem from a movement impairment. Unusual patterns of movement have been observed in autism since its first descriptions, and motor coordination difficulties have been found in up to 80% of autistic individuals (Fournier et al., 2010; Gowen and Hamilton, 2013), yet, with the exception of repetitive movements, motor symptoms are usually treated as being peripheral or additional to autism (Leary and Hill, 1996; Ming et al., 2007). ‘Autistic behaviors’ such as non-compliance, lack of communication, lack of affect and resistance to change could be due to difficulties with initiation of movement. Ming et al. (2004) reported this phenomenon in a single case of an adolescent autistic girl who was considered severely non-compliant. The participant was instructed to squeeze a hand grip while muscle action and physiological correlates of mental effort were measured. Indications of internal effort were seen even when no muscle action was recorded. Further evidence of a mismatch between intentions and actions comes from analyses of the memoirs of three minimally verbal autistic young people (Welch et al., 2018) as well as the writings of autistic bloggers (Welch et al., 2020). Both of these papers included several themes related to initiation problems, e.g., ‘I can’t start my body’ and ‘Brain-body disconnect.’

Slow movement and delayed initiation have been reported in autism since the mid 1990s (Leary and Hill, 1996). This was first detailed in the context of catatonia in 2000 (Wing and Shah, 2000). Catatonia, a complex psychomotor syndrome, is typically envisaged as a lack of responsiveness to the environment (stupor) and freezing in awkward positions (posturing); however, Wing and Shah described a range of difficulties including extreme slowness, freezing mid-movement, prompt dependence, repetitive movements, mutism, and deterioration in self-help skills. The full catatonia syndrome occurs in up to 20% of autistic people (Wing and Shah, 2000; Billstedt et al., 2005) and nearly half of a group of 87 autistic adolescents were found to have clinically significant catatonic features (Breen and Hare, 2017). As with cognitive flexibility and repetitive behavior discussed previously, catatonia may be associated with abnormalities in thalamocortical loops although the exact mechanisms have yet to be clarified and there are indications of diffuse pathway dysregulation (Daniels, 2009). Wing and Shah expanded on their description of catatonia in autism in a paper exploring catatonia-like features without marked deterioration in autistic children and adults (Wing and Shah, 2006) and numerous cases are detailed in Shah’s recent book on the subject (Shah, 2019). In the literature, the most extreme forms of these motor issues are usually associated with ‘severe’ autism, it is now understood that catatonia can have a range of expression from the most recognizably severe manifestation to mild and intermittent. It is possible, therefore, that more subtle expressions of catatonia are under-reported within the autistic spectrum more widely.

Despite the high prevalence of catatonia-related phenomena and the severe impact on functioning, autistic catatonia has been under-explored in research. Moreover, due to the severe disability of those who have been studied, what research exists has been based almost exclusively on second-hand reports from carers and clinicians. However, milder expressions that would not be readily recognized as catatonia may share some underlying characteristics. There are obvious limitations to the understanding that can be gained by observation of a condition characterized by impairments in action and expression. Welch et al. (2020) explored autistic embodiment through analysis of blog posts by both speaking and non-speaking autistic people. They found some difficulties regulating movement which were reminiscent of catatonia-like impairments. While this work contributed to the understanding of internal autistic experience, those who experience the most significant impairments in voluntary action are unlikely to be able to consistently write a blog. The present study further explores these issues, focussing specifically on the ability to act on intentions, by talking to autistic people who share some of these difficulties. In particular, characteristics that are often invisible to observers such as difficulty initiating, emotional states, and motivational factors may be clarified by first-hand reports.

In summary, inertia is commonly reported by autistic individuals, but has not previously been the focus of any formal research. In order to explore the nature, mechanisms and impact of inertia, this study used first-hand descriptions, collected via focus groups, of difficulties autistic people experience with doing things they need or want to do and their impact on day-to-day life. This is an important topic for the autistic community, with implications for our understanding of and approach to a subset of autistic behavior that creates challenges both for caregivers and for autistic people themselves. The lead author’s personal interest in the subject inspired her to attempt to assist autistic people suffering from these difficulties on an individual basis as well as organizing informal discussion groups on ‘autistic inertia’ and ‘catatonia’ at Autscape (an annual residential event for autistic people) in 2017 and 2018, respectively. Each of these groups was attended by approximately 40 autistic individuals who shared their experiences and provided mutual support. Participants in these groups strongly advocated for research into the subject, and these discussions have informed this research.

## Materials and Methods

### Approach

The aims of this research were to explore the experience of inertia and to begin to describe these experiences and their impact. So little is known about this collection of difficulties that a broad approach was required and a realist framework was adopted for this initial description. Following the success of previous discussion groups at Autscape, which gave participants an opportunity to share their experiences and hear those of others, data was collected through focus groups, both face-to-face and online. Previous research on related topics has used only observation, second-hand reports from carers and writings of autistic people, so first-hand reports allowed for unique insights into the internal experience of autistic inertia. This study was approved by the University of Manchester Research Ethics Committee (ref. 2019-6324-11577).

### Recruitment

The face-to-face focus groups took place at Autscape, a well-established annual residential event in the United Kingdom organized by and for autistic people. Autscape does not allow researchers to approach potential participants to avoid pressure or coercion, so recruitment was entirely by the placement of posters and sign-up sheets for the group sessions. The sample was purposive; the recruitment posters referred to experiences of getting stuck or having difficulty doing things. Despite the restrictions on recruitment methods, two additional groups were needed to accommodate the high number of volunteers.

Two further online (Skype) focus group sessions were conducted because many autistic people have difficulty with travel to unfamiliar places and interaction in groups. Text rather than video chat was used in order to maximize access because autistic people often have difficulty with various aspects of social communication such as the timing of conversation turns, auditory processing, and attention. Several adjustments were needed to improve accessibility to autistic people, for instance by requesting that moving images not be used to reduce the visual processing stress. More detail about conducting text-based meetings with autistic participants can be found on the website of autism research charity, Autistica (Buckle, 2020) and the Autism@Manchester website (Buckle and Gowen, 2021).

In order to obtain the widest representation possible, the selection criteria were kept to a minimum, with no exclusions for psychiatric or other conditions which commonly occur with autism. The requirements were that participants must be age 18 or over, clinically diagnosed with any autism spectrum disorder and able to express their experiences in words.

### Participants

Informed consent was obtained from all participants prior to engagement in the study. Demographic data were obtained with a brief written questionnaire. Participants consisted of 32 adults age 23–64 years (mean = 45). Their self-described genders were: 19 female, 8 male, 4 non-binary, and 1 unspecified^2^. All had a clinical diagnosis of any autism spectrum condition by a suitably qualified clinician or multi-disciplinary team. Where possible (*n* = 14), diagnosis was verified by having sight of the participant’s diagnosis letter. Where the diagnosis letter could not be obtained, details of the diagnosing clinician, clinic and date were taken.

The remaining background questions, which were not answered by three participants, are reported here in order to fully characterize the sample. All but one participant (who lived with their parents) lived independently in the community. Half of the remainder (*n* = 14) lived alone. Of those who lived with others: 4 with a partner, 3 with their children, 6 with partner and children, and one with a flatmate. 18 individuals received care or support due to their disability. Nine of those who responded worked full time, 7 worked part time, 4 were students, and 9 were unemployed or retired. Participants reported the following mental health diagnoses: 24 had anxiety, 20 had depression, 12 had been diagnosed with PTSD (5 had recovered), 2 had a past diagnosis of psychosis, but one considered this to be a mis-diagnosis before their autism was recognized. Eight reported that they were taking neuroleptic medications or had in the past. They also reported the following neurological conditions: 8 ADHD, 7 dyspraxia, 8 migraine (not asked on form) and one had a diagnosis of catatonia. Additionally, 4 reported a fatigue-related condition such as chronic fatigue syndrome.

### Procedure

Six focus groups were held, each attended by 4–6 participants. Details of the composition of each group are given in Table 1.

**TABLE 1:** Focus group composition.

Group	Format	*n*	Age mean (range)	Gender		
				Male (*n* = 8)	Female (*n* = 19)	Other (*n* = 5)
1	Face-to-face	4	46 (32–64)	2	2	0
2	Face-to-face	6	49 (37–62)	0	4	2
3	Face-to-face	5	43 (33–53)	2	2	1
4	Face-to-face	6	45 (36–51)	3	3	0
5	Online	6	44 (25–58)	1	4	1
6	Online	5	45 (23–45)	0	4	1

*Other includes both non-binary and unspecified gender*.

Four face-to-face groups were held over the 3 days of the Autscape event in July 2019, and a further two groups were held online in May 2020. Online focus groups were conducted after the data from the face-to-face groups had been collected and analyzed. The lead author (KB) conducted all 6 groups, and participants were made aware that she is also autistic and experiences significant difficulty with initiation. In addition, one online focus group was attended by KL and the other by EG. Each meeting lasted 1.5–2 h. The initial half hour provided an opportunity for participants to familiarize themselves with the research and procedure for the session and to ask any questions about what would happen. Participants were encouraged to talk about their experiences of difficulty doing things they want or need to do. Questions were oriented around ‘difficulty doing things’ because the researchers anticipated from background understanding that these may not be easily segregated into difficulty initiating a task vs. difficulty stopping an ongoing activity in order to initiate a new one. Questions were asked according to a schedule to prompt a range of responses (Table 2). Any participant who had not contributed was specifically invited to do so before moving on to the next question, with an explicit option to pass. This was rarely needed and nearly all participants responded to all questions. Face-to-face groups were audio recorded and later transcribed by a professional transcription service.

**TABLE 2:** Focus group questions and prompts.

What are some experiences of difficulty doing things?
*Prompts:*
• Do you have any specific examples of when you’ve been unable to do something you needed or wanted to do?^*a*^
• Are they things you want and are motivated to do?^b^
**What makes it harder?**
• What do you think stops you from getting things done?
*Prompts:*
• Do you get paralyzed with anxiety?^b^
**What makes it easier?**
*Prompts:*
• Plans, schedules, or alarms^b^
• Someone else starting it off^ b^
• Does music have any effect?^a^
**Can you describe what it feels like to be ‘stuck’?**
*Prompts:*
• Do you feel like you’ve slowed down (or everyone else has speeded up)?^b^
• Do you feel anxious?^b^
• Do you know how much time is passing?^b^
**Does this have an impact on your life?**
*Prompts:*
• For example, your ability to be productive – study, work, parent, volunteer, etc.^a^
• Your ability to take care of yourself^ b^

*The main questions (in bold text) were asked of each group. Prompts were only used if no one in the group spontaneously mentioned the topic. ^*a*^Prompts that were used in most or all groups. ^*b*^Prompts that were used in a minority of groups*.

### Analysis

All face-to-face interviews were completed prior to transcription due to time constraints at the Autscape event. Text transcripts of the audio recordings were carefully checked against the recordings for errors or omissions. The text from the online groups was used as written by the participants with minor corrections of punctuation and spelling. Data about gender, age, support needs and co-occurring conditions were collected in order to fully characterize the sample, and to indicate possible avenues for further research. Additional diagnoses were not verified. Therefore, in this study, data were not analyzed separately according to additional conditions.

Data analysis was conducted using inductive thematic analysis, following the reflexive method set out by Braun and Clarke (2006, 2019, 2020). Because the aims were concrete and descriptive, a realist framework was used in which the experiences of the participants were coded and interpreted on a semantic level, without reference to social context or unarticulated meaning. After familiarizing herself with the data, KB exhaustively applied codes to each concept present within the data. The codes and categories were reviewed, analyzed, refined, categorized, and combined to generate themes and group them in meaningful categories. This was an iterative process and developing the structure to include a manageable number of themes required reanalyzing the codes, categories and themes several times. Saturation was reached after five groups, with no new themes arising in the final (sixth) group. Coding was conducted only by KB, who then discussed and refined themes in collaboration with all authors, with a selection of participants (described below) and again following peer review.

KB made an effort to reflect on her lived experience and prior exploration of autistic inertia, and the influence this would have in analysis. In particular, KB had a pre-existing belief that autistic inertia cannot be explained by anxiety alone and there is a movement component to initiation difficulties experienced by some autistic people. KB also has influence as a leader within the Autscape organization which may have affected participant responses; however, this background also contributed to trust and rapport within the groups. A visual record of the development of the themes was maintained in order to review decisions and confirm that important concepts had not been lost.

#### Validation

A selection of participants were consulted throughout the analytic process, which helped to shape the themes and final structure. The results were presented to many of the participants and others following Autscape 2020. Participants confirmed that the analysis was an accurate representation of their experiences. One responded that the experiences described were so close to their own that they could not readily identify which quotations were theirs. Another said that reading about others’ experiences helped him to be more forgiving of his own difficulties. Participants approved of the theme structure and analysis without any requests for corrections.

## Results

**FIGURE 1:**
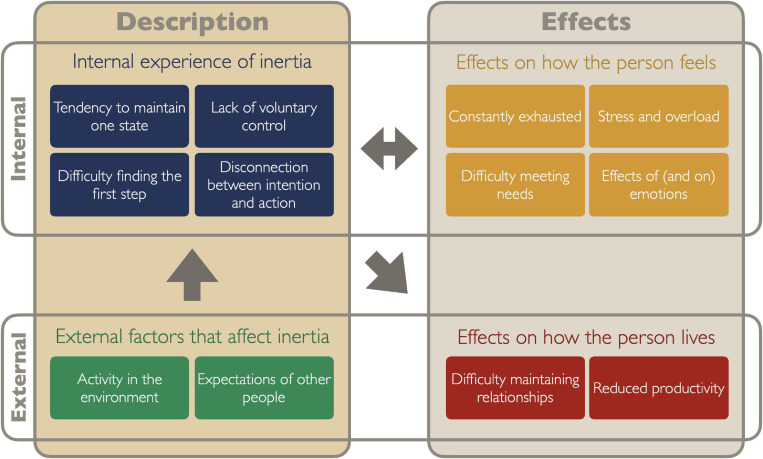
Diagram of themes in autistic inertia. Themes (colored rectangles) are organized into primary categories (columns) of ‘Description’ and ‘Effects.’ These are further divided into internal and external domains. This then provides four sub-categories: internal experiences of inertia (blue), external factors that inhibit or facilitate action (green), effects on how the person feels (yellow), and effects on how the person lives (red). Arrows indicate the direction of effects as one area influences another.

The present study investigates autistic people’s experiences of difficulty doing things they need or want to do. Topics arising during focus groups that were not related to this (e.g., general attitudes about autism and experiences of the Autscape event) are not included. The autistic community jargon of ‘inertia’ was often used to refer to difficulty stopping, starting and changing tasks. A diagram of the themes is provided in Figure 1. Participants related their experiences objectively and analytically, with honesty and candor. They provided detailed descriptions of their difficulties in considerable depth, and these fell into two main categories: descriptions of inertia itself, and its effects. Within each of these categories, four themes reflected internal experiences and two themes related to how inertia interacted with the external world. Each of the themes is described with illustrative quotations from the data. Quotations are provided verbatim and names are pseudonymised to protect privacy.

### Descriptions of the Internal Experience of Inertia

Four themes described core characteristics of participants’ internal experiences of inertia: tendency to maintain one state, lack of voluntary control, difficulty finding the first step and disconnection between intention and action.

#### Tendency to Maintain One State

Participants were asked about problems ‘doing things’; however, in their responses, difficulty acting encompassed not only starting, but also stopping and switching tasks.

*I can’t get to the point where I’ll go to do the thing because it’s almost like I got to stop whatever I’m doing, whether I’m doing anything or not. Even stopping not doing anything is stopping doing something. – Ruth.*

Because of this difficulty differentiating starting from ‘stopping not doing anything,’ and true to the ‘inertia’ metaphor, the term ‘inertia’ is used to refer collectively to these related functions, especially when these interfere with initiation of new activities.

Continuity of a task made it easy to stay in the same state of activity or inactivity; interruptions would disrupt that, which could be helpful or harmful depending on whether the person wanted to switch. Several participants stated that starting was more problematic than sustaining action, e.g., Ruth said, *“I’m alright once I get going but getting going can take a long time.”* As an indirect consequence of difficulty stopping, they would sometimes avoid engaging in certain activities for fear of being unable to break away when they wanted to.

Once inertia was ‘in motion,’ any disruption could derail a task completely. Participants were unable to suspend a task for an interruption and pick it up again, which at times made them reluctant to even begin. Similarly, having to interrupt a task to do something else, such as to fetch a necessary item, would then make it very difficult to re-start the task.

*I’m in the office and there’s paper everywhere and I’m trying to sort it out. And then I do have to go and do something else, so, you know, the child needs to eat food or whatever. Like I just can’t get back to the place I was before. So, I can’t get back to the task. And then it’s even worse than it was before. The fear of that happening stops me from starting a new task. – Elizabeth.*

A break in continuity, such as any kind of barrier, could make a task impossible to initiate or sustain. The barrier could be (a) physical, such as an item blocking access to the washing machine; (b) social, such as having to walk in front of another person; or (c) psychological, such as having to make a decision:

*I spend the whole day not quite deciding whether to have the shower first or do something else first or do a load of laundry, and then maybe go out after doing the laundry or go and get it over with. – Daniel.*

Perfectionism and a desire to make the ideal choice exacerbated these issues.

Conversely, strategically-placed barriers and visual cues could interrupt a habitual action and make it easier to engage in a desired activity. For example, placing an instrument and music in the way of getting from one room to another. At a more basic level, an interruption could trigger action for someone who was frozen.

*Sometimes, I end up just sitting and not doing anything when I really want to be reading a book that’s right next to me, but I’m not. And that can last often till there’s an external sort of interruption, which is normally like if my partner walks past the door, then I’ll be like, ‘Oh, I should move now.’ – Lisa.*

Difficulty starting occurred most often with procedural barriers, such as having to put on shoes, or where a sequence of actions was needed:

*There are always things that need to be done, before I can do ‘the thing’ – so, I want to brush the hall floor (not sure why) but first I need to move things from hall floor. Move crutches… where to? Somewhere accessible, but not in the way – sidetracked. scissors picked up – oh, now I’ve found scissors I can… Sewing machine still there after 5 months – need to put it away, but there’s no space for it and my son tidied my shed, so must keep it tidy. Exhausted, lie down! – Harriet.*

Such barriers typically not only blocked the task they were trying to do, but also transitions to another task. The bigger the barrier, the more difficult it was to start; more significant changes of position (e.g., getting up) or transitions (e.g., leaving the room or house) were more problematic than starting tasks that didn’t require such transitions.

*It takes me an awful lot to get out of the house. There’s so much that has to be done to get out of the house at vaguely the right time with all the stuff that you need and properly dressed and so on. And I actually find if I can put all that preparation in – which is a lot of preparation – for all the things today and go out and not come back home again so it’s done and go from one activity to another activity to another activity. It actually takes a lot of the pressure off because the hardest thing for me of all is leaving the house. – Nicky.*

Strategies used to overcome these issues often involved reducing barriers, lowering the threshold to action. Several participants talked about getting themselves to start something by consciously breaking it into small steps and talking themselves through it. For example, when having difficulty getting out of bed:

*Eventually saying to myself, ‘All you need to do is….’ And so, all you need to do is sit up, sit on the bed. All you need to do is sit on the edge of the bed, stand up, you know, walk out of the bedroom to the bath, et cetera. And then I suppose similarly getting dressed – Fred.*

Sometimes they would deliberately avoid stopping an activity because they feared being distracted or becoming stuck in an inactive state, and unable to return to their original activity.

*I find sometimes I have to say to myself ‘Don’t sit down, don’t sit down, don’t sit down yet. Okay, now you can sit down.’ – Emma.*

Although the focus of the discussion was on the difficulties of not being able to do things they need or want to do, the tendency to keep going could help to get tasks completed or was desirable in itself. These were to do with the ability to, as Paul said, *“become totally immersed in some things.”* This could be specific to the task at hand, or a more general feeling:

*I’d like to mention the flipside to all of this when everything goes right. I do everything with extreme capability, and everything is just right, and that other thing that’s nagging, pestering at the back of my mind is not present and it’s like it takes no time. Everything feels like it’s so fast and you do everything so quickly. – Joel.*

Deep immersion in a desired activity was described as pleasurable, even if it involved losing track of time, again as Joel contributed, *“When I’m focused on something, I’m not aware of how much time’s passed. It could be an hour. It could be 6 h.”*

#### Lack of Voluntary Control

The other central aspect of their difficulty was that it was experienced as involuntary and impervious to their conscious efforts. For example, Margaret wrote: *“I also can’t overcome my inertia. I have to wait for it to go away.”* Despite the insightful and detailed descriptions of the problem, many felt that their problems were unpredictable and largely incomprehensible, describing them as ‘ridiculous’ and ‘illogical.’ In particular, they were confused by their inability to execute tasks that were within their capability, and by the variability of both the expression of the problem and the effectiveness of strategies.

*Yes, I never manage to schedule cooking for the week, but often when I do manage to cook I make several meals worth. It’s just I can’t guarantee to manage that when I need to. – Naomi.*

This effect was quite noticeable in the difference between an established routine and spontaneous action.

*Routines. Routines help a lot. Anything that I can routinise so that I don’t have to think about it quite so hard helps. And then I can do those things a bit more on autopilot. – Lisa.*

Conversely, a plan that required internal effort to execute could be impossible, despite sometimes acute awareness of the consequences:

*I think it’s the expectation as well. Like for example, I know I can’t switch between tasks during the week, so I decide to cook all my meals on Sunday and meal plan. But then Sunday comes and the pressure of doing this thing in that specific timeframe is really too much, so I struggle to do it because of that. Instead, I spent the day panicking and being mad at myself for not doing it and thinking of how much it’s going to ruin the rest of my week. – Margaret.*

They reported having to manipulate themselves into doing things.

*I just don’t feel like I have control over what I’m doing necessarily. I feel like I’m coaxing myself through things or I’m trying to work out strategies to make myself do things. – Alex.*

They often tried to convince themselves to act, but this was usually ineffective:

*My head is saying all the right things, like you’ll feel better if you do X, or if you get up now, you’ll be able to do that thing you’re excited to do, but it’s like the rest of me is a stubborn child… I think there’s some demand avoidance in there too. – Jo.*

Together, the tendency to stay in the same state and the lack of voluntary control were threads that ran through descriptions of inertia and the factors that influenced it.

#### Difficulty Finding the First Step

Several participants felt there were different issues underpinning their difficulty acting, for example:

*It’s almost like there’s two different kinds of things. It’s kind of like there’s the kind of the mental kind of plan-y sort of stuff, which is more kind of stress-inducing almost. And then there’s a different kind […] almost like a physical thing where you just kind of get physically stuck. – Ruth.*

Difficulties with planning, and with executing a plan, were a major issue. Some had difficulty breaking down a task or formulating a plan:

*Too many different things need doing – can’t prioritize. Very easily overwhelmed by amount/number of things to do. […] Difficult to separate the ‘blob’ of ‘lots to do’ into small, potentially doable bits. - Harriet.*

Some had difficulties that were almost the opposite. They could break down a task, but continued to break it down until it had so many competing elements they were unable to see how to proceed:

*I can cook. But a lot of the time, I buy the ingredients and I never cook anything because it gets too complicated in my mind. – Joel.*

Others expressed difficulties to do with prioritizing or finding a starting point. They often needed the help of another person to work out how to approach a task.

*It’s having a tornado of things going through your head, trying to work out how to focus on one thing and work out how to pick one thing. Some might be tasks to do (fun or not), some will be processing the day or specific info, all of which makes it harder to find a way in to the ‘to do’ things. - Jackie.*

A weak working memory could create issues with planning and with executing any plan. Alex relates it like this, *“I will go ‘oh, I’m in this room now, what was I doing? I was doing this thing, I’ll go and do that – oh no, apparently, I’ve done that already.”* Alex could forget whether they’d used their toothbrush, whether they’d taken a puff of inhaler seconds earlier, or even whether they had already made a decision.

#### Disconnection Between Intentions and Actions

In addition to planning difficulties, a subset of participants also felt, at times, that there was a disconnection between their perception, emotions, intentions and actions. Unlike the difficulties related to planning and prioritizing, which tended to affect complex tasks, disconnection between intentions and actions could apply to actions that seemed simple. Thomas said that, *“It seems ridiculous sometimes. You just can’t do certain things that seem so simple,”* and Lisa provided an example:

*For some things like I find it really difficult to work out why I’m not getting started especially when it’s something I really want to do. […] and there are only one or two steps for me to start doing it like picking up a book that’s right next to me. I just don’t… I don’t understand why that’s so difficult sometimes.*

This experience of disconnection had three distinctive characteristics: feeling physically unable to move, altered awareness, and passivity.

##### Physically unable to move

Participants described their experience as ‘physical’ and that although they knew what to do, they ‘just can’t.’ Although analysis was not conducted with *a priori* codes, it was recognized that this description had several characteristics in common with catatonia, although sometimes with a more subtle expression. Examples of these are provided in Table 3.

**TABLE 3:** Examples of catatonia-like features from participant reports.

Catatonia	Example of related experience from focus groups
Periods of shutdown^a^, being very still for long periods of time^b^	*I’m going to make a drink and standing and then realizing an hour later that I’m still standing in front of the kettle and haven’t actually done anything. I’m thinking but why, how has that happened? - Sam*
Movement difficulties (freezing and getting stuck)^a^, getting ‘stuck’ when trying to complete actions^b^	*It feels like I’m holding my breath and my body is frozen. It’s a literal inertia. I mean it’s a literal paralysis and very often I will find that I am actually holding my breath… So the feeling of it is literal, nothing moving, no thought, no breath, no movement. - Emma*
Difficulty stopping actions once they have been started ^b^	*I find one of my things is reading news websites now, and that I just end up reading sometimes different articles and sometimes sort of the same one over and over. - Lisa*
Difficulty initiating actions^b^	*I’m finding it really difficult to actually just pick up a book and get started. - Lisa*
Increased slowness^a^, moving very slowly^b^	*I feel I can move but like really slowly and only to like lie down or curl into a ball as I feel frozen or freezing up. - Kelly*
Prompt dependence^a^, taking a long time to finish actions or requiring prompts to complete actions^b^	*If I’m struggling to get to bed and my partner has already gone to sleep, chances are at some point, he might get up to go to the loo or at least I know at some point he’s going to get up in the morning. And that will probably unstick me… - Lisa*
Movement abnormalities^a^	*When I put the washing machine on, I find I spend an hour in the kitchen […] kind of swaying around or juggling or just doing things […] And somehow, that washing machine, when it’s on it’s like I’m magnetized into the kitchen. - Daniel*
Passivity and apparent lack of motivation^a^	*If I’m wanting to do a social thing, I’d like to spend time with people, I have difficulty initiating […] I wouldn’t even sometimes think to contact them to start it. I’ll just go, I would have liked to have been doing something with somebody and it’s been quite a new thing to realize that I can start a conversation with somebody like somebody else doesn’t have to start the conversation first which isn’t always evident. - Ruth*
Posturing^a^	*It’s like im frozen in time… at worst it can hurt cause it feels like I want to move but can’t - Brian*
Fluctuations of difficulty^a^	*I just find myself utterly baffled when I’m just stuck. And sometimes, I’m stuck for days on end and just in the contrast with how productive I can be on other days. It just baffles me. – Elizabeth*
Catatonic excitement^a^	Not evident in the data.

*Characteristics of autistic catatonia drawn from two sources, as indicated: ^*a*^Primary difficulties and manifestations of autistic catatonia (Shah, 2019, p. 29). ^*b*^‘Core’ features of catatonia in autism from Attenuated Behaviour Questionnaire (Breen, 2014)*.

Some participants found themselves unable to take a specific deliberate goal-directed action while still being able to move in other ways. For example, Daniel would struggle to get out of bed, despite being thirsty and needing to use the toilet, even while he could play with his phone.

*Sometimes, a drink is actually… maybe not in arm’s reach, but in like standing up a little bit and reaching, reach. I can’t understand why I won’t get it. And afterward, I think… how was that about? Why did you give yourself a headache and do that for 45 min or something until you’re almost on the verge of wetting your bed? And then, what’s going on? Am I myself when I’m doing that? Is somewhere the physiological thing taking over? What is the problem? … It’s not an every day thing for me, but […] it usually eventually results in pain, dehydration headaches, things like that. – Daniel.*

##### Altered awareness

In addition to describing physical difficulty moving, participants talked about their internal experience during such episodes. Sometimes the person felt disconnected from their physical body, thus unable to control it.

*Sometimes when I feel stuck, if it’s… where I feel I can’t for example get off the sofa, it’s almost that dreamlike state where I can hear everything but it feels kind of slightly far off. – Suzanne.*

Sometimes they felt ‘stuck in their mind,’ unable to enact things with their body:

*I can’t unpick what I need to do, where to start or how to find the energy to get beyond the thinking about things… and then 3 h just goes out the window - Brian.*

This could be combined with altered time perception, for example:

*I am aware of my surroundings, but time feels slower, more drawn out and I don’t remember being able to feel my body other than being frozen but it feels as if I go completely into my head, like an out of body experience but in my mind. – Kelly.*

At times, they could even experience a complete cessation of thought, awareness and action, so the person would find themselves in exactly the same position, but between several minutes and several hours had passed.

*Sometimes, I’d be like, ‘Oh, I want to read. Oh, here’s a book.’ And then I’m reading the book. And sometimes, I’d be like, ‘Oh, I want to read.’ And then it’s 3 h later and I haven’t moved. – Lisa.*

##### Passivity

Even while conscious of the inability to perform a desired action, there was minimal sense of physical or mental strain. They seemed to simply accept that the desired movement did not happen.

*I’ll be sitting on the bed thinking I should really go to bed. I really want to go to bed. I’m really tired. But it’s just not happening, but it doesn’t worry me, the way that sometimes things really worry me. – Lisa.*

Like Lisa, others often had little emotional arousal about the situation.

*It feels like what I’m thinking is sort of somewhere out here, kind of passively observing myself and going, ‘hmm. I’m not actually doing that thing that I want to do. I’m not sending the text message I could be texting. I’d quite like to contact the person.’ So, I’m not like stressed about it or anxious. … it’s like commentating on it but in very sort of, ‘oh, that’s mildly interesting’ sort of way. – Erin.*

### Descriptions of External Factors That Affect Inertia

In the absence of internal drive, participants found themselves dependent on the scaffolding provided by external cues and prompts.

*Like I am stranded in the middle of the sea and nothing exists anymore. There is no past, no present, nothing to do and no way out except from external intervention. – Margaret.*

The key external factors, activity in the environment and expectations of other people, could both facilitate and inhibit action.

#### Activity in the Environment

Several participants described human and non-human elements of their environment prompting and sustaining action. Environmental cues, like an office environment, and synchronous activity, such as someone working on a similar task nearby, could help the autistic person to do the same.

*Sometimes, what helps me is having another person present, but I don’t necessarily want them to interact with me. Just there working beside me, maybe doing the task with me, but not… just working side by side just kind of motivates me for some reason. – Daniel.*

Conversely, asynchronous activity or irrelevant movement and background noise was usually distracting and stressful. This was most pronounced in the highly varied responses to music. Some found that music put them in the mood to act and could make it easier, while others found it a problematic distraction.

#### Expectations of Other People

The most often reported helpful factor was the assistance of another person; however, the influence of others could also hinder action if it was stressful or demanding. Several participants said that prompting by another person could be very helpful for getting unstuck.

*The only thing that helps me, only thing that works, and it works consistently, is just to have a stuck buddy that I text. … And all I have to do is text, ‘I’m stuck.’ […] And we just text it out and kind of make a plan. – Elizabeth.*

Being expected to do something for or with someone, such as by scheduling an activity with another person or being needed, was often helpful:

*It’s much easier to do something for another. I can even do form filling with another person and I’m hopeless with forms. So for somebody else then yeah, that makes me do it. – Nicky.*

The most effective supports were time sensitive. A sense of urgency could make even stalled actions possible for some participants:

*Sometimes having to do something straight away helps. Once a friend asked for a cake recipe but kept saying no pressure, when you’re ready, and I failed and failed to send it for weeks, Then 1 day she emailed and said she needed it for tomorrow when she had visitors coming and I just did it straight away! After all those weeks. – Naomi.*

Some participants recognized this and deliberately scheduled external time-sensitive activities such as having to be somewhere at a specific time to open the room for a group meeting or attend an appointment:

*If there’s things I need to do like there was giving blood a while ago, I had to schedule a time to go, then I deliberately got it sort of 8:30 in the morning to get myself out of bed. So I had to go there. And then sort of just getting myself out, forcing me to have breakfast and get there and I then find the rest of the day so much better than if I sort of don’t have something to force me up relatively early. – William.*

Deadlines had a similar effect of helping some people to act, although the combination with stress meant that this had a cost:

*I’ve been in a lot of situations where I pretty much don’t have a choice. Like I’ll either complete this by the deadline or I’ll be homeless sort of thing. That pushes me through, but also makes me live in a constant state of fear. – Margaret.*

For others, like Brian, the stress from the obligation outweighed the prompting effect, so that *“a deadline really doesn’t help. If anything it makes it harder to start.”*

External expectations of another person could not be easily substituted by artificially created structure or urgency using electronic or cognitive strategies. Lists, reminders and alarms were helpful for some; however, when asked if alarms were helpful, Harriet’s answer, *“Not really – reminds me I have to do something, but doesn’t help me overcome the inertia,”* was typical, as was finding the suggestion laughable. Joel reported that there was *“a big difference between having a support worker and having no support worker,”* and Sam felt the only thing that could help them do things would be another person:

*I’ve pretty much tried everything. We’ve tried all of that. And it just doesn’t, and that’s great if it does help for some people but it made me feel there’s no hope really. I think unless I have a physical person helping me do these things. – Sam.*

### Effects on How the Person Feels

General wellbeing, such as energy levels and mental health, both affected and was affected by the participants’ initiation impairments. Participants frequently reported being constantly exhausted, stress and overload, difficulty meeting needs, and the effect on (and of) emotions.

#### Constantly Exhausted

Participants frequently reported states of fatigue which made it harder to act:

*My validation has come from my diagnosis which recognized how my extremes of anxiety, uncertainty, executive functioning and SPD [sensory processing disorder] mean I am constantly exhausted. – Jordan.*

William found painful emotions so draining he could be unable to act:

*I find that sometimes thinking about it then makes me tired because it’s […] very painful. But actually, just thinking about it makes me really tired.*

Several participants also needed extended periods to recuperate:

*It’s perfectly valid to me that I’m not doing stuff because I know I’m too tired to do it. I know that it would be stupid to leave the house at this point. I know that it would be ridiculous, I have no spoons^3^, as they say, so why am I thinking that I have to do things? […] So I try to be decent to myself….*

*I know that what I really need is what I call kind of 3-day recoup … in which I basically crash for 3 days, watch television, do nothing except eat and watch television and zone out and doze off and stuff. – Emma.*

#### Stress and Overload

Participants talked about stress, both from their lives in general and from the tasks they struggled with. Jackie talked about *“getting so overwhelmed that I can’t speak, can’t move. My head is just working overtime but I can’t actually get any words out.”* Often stress was related to sensory aspects of the task or environment, even simply the requirement to move their body.

*When people talk about sensory overload, most people assume that just means what they consider to be the main basic senses. I don’t think they take into account that stuff such as being too hot or being in too much pain or just being too tired. I don’t think a lot of people appreciate that, that just those things can be so overwhelming that it’s that difficult to do anything else. – Suzanne.*

Stress reduction strategies made things better. Some talked about the benefits of being outdoors or listening to or playing music in order to put them in a better place to approach a problematic task. Stress featured in all of the factors that made initiating more difficult.

#### Difficulty Meeting Needs

While poor wellbeing made it more difficult to initiate, failing to do things in turn negatively affected wellbeing, creating a self-perpetuating cycle. As described earlier, even basic needs such as drinking or going to the toilet could be left unmet until they became desperate. In addition, many participants described difficulties with exercising and carrying out self-care routines, which affected physical and mental health:

*It can also affect my ability of looking after myself sometimes. Showering often enough. Doing my teeth was a massive thing, rarely did them cause I hate the sensory and just process of doing it. Having actual reasons to do things makes a big difference. Just ‘looking after yourself’ doesn’t tend to be a good enough reason. – Helen.*

Living conditions also suffered, including difficulty cleaning, clutter, and problems with household tasks. One described her house as a ‘constant mess.’ Another described various difficulties with maintenance of essential household facilities.

*I’ll be meaning to buy a tumble dryer for the 5 years I’ve lived in a house. I’ve still got paint samples on the wall from when I moved in. My shower was unusable for a year, and I’m lucky to have a bath as well, which doesn’t work properly. So, bath’s been with a bucket and stuff. – Daniel.*

#### Effects of (and on) Emotions

Strong negative feelings prevented participants getting started on tasks. These included anxiety, painful emotional content, and depression.

*Lots of the things which I have difficulty would seem to be anxiety driven and based around perfectionism as well. […] For me, it’s easier if it’s something which I actually feel more comfortable with anyway which is why I think it is anxiety driven. – Suzanne.*

However, several participants were clear that although they had anxiety, their inability to do things occurred whether they felt anxious or not:

*Even if I feel totally relaxed and happy, you know, some days, I can’t formulate the plan so I don’t go out at all and that happens once or twice a week. So that is very disabling. – John.*

Depressive thinking made Alisha feel she didn’t deserve to take care of herself; however, in the light of her recent understanding of autism, she said, *“I wonder these days […] was that depression or was that just this unknown thing that kind of… I’ve never been able to…”*

Avoidance of anticipated negative emotions was as important as being paralyzed by overwhelming emotion in the moment.

*But the other thing is just that that fear of making it even worse. Because every time I try, it seems to be it seems to end in failure rather than success. And it’s just that constant feeling of I messed up again. – Elizabeth.*

Emotional factors were mentioned more often as a consequence of failing to act than as a cause:

*Anxiety makes it worse, for sure, but I often also have anxiety about not getting things done that I really need to do. – Naomi.*

Many participants were able to accept, most of the time, that their inability to act was outside their control, but nearly all expressed frustration as a result. Some felt considerable guilt and inadequacy for their failures, which damaged confidence and made them feel hopeless. This, in turn, made it more difficult to act. Having an understanding that this problem was not something they could control helped considerably.

Sometimes when unable to act, a participant would just get on with something else, while others would be apathetic. The fact that things were not getting done and time was getting shorter would itself often cause frustration, even when the inability to move did not, as discussed in “Disconnection Between Intentions and Actions.”

Participants expressed that other people often did not understand the magnitude of their issues. Others would assume that the failure to do a task was due to forgetting and would offer trivial solutions such as alarms and reminders.

*People are like, ‘well if you just do this, if you set a reminder if you do whatever,’ but it’s like you have no idea. Like you’re so far away from the truth of my existence. I feel like, you make me feel like I’m lying and I end up starting to question my own truth. And I know it’s true. – Ruth.*

Some were aware that others might see them as lazy or not trying, but recognized that they could not do better.

*I know that even if externally to people watching me, it might look like I’m not trying, but I feel like if I’m not getting the stuff done, I know like for myself that it’s not just because I’m lazy or not trying. It’s just because I can’t cope with it at that point, and I can’t do it. – Lisa.*

### Effects on How the Person Lives

When asked about the impact of initiation impairments on their lives, several participants answered that it affected ‘everything.’ This included both things that they wanted to do and things that they needed to do. Some referred to inertia having a general deleterious effect on their quality of life.

*I think I kind of sum it up on my life is probably a lot smaller and less than I would like it to be. Just in general, there’s a lot less in it. I would like a bit more in it, but I don’t have the ability to make more in it. – Sam.*

In addition to the effects on general wellbeing discussed above, there were two further sub-themes describing effects of initiation impairments on their activities: reduced productivity and difficulty maintaining relationships, which were both touched on by Catherine, who said, *“I cannot work and have friends and maintain the house all at the same time. I just can’t do it.”*

#### Reduced Productivity

Participants reported that their difficulty doing things affected all areas of productive life; in some cases, the inability to act was the main barrier to employment:

*Just my ability to earn money and not relying on the state. And it’s just the frustration of, and people meeting you and being like, you’re really eloquent and whatever. And it’s like so what? It doesn’t translate to an ability to utilize that in the world in a way that makes me enough money to live independently and do the things I want to do. – Ruth.*

Some found that work was the only thing they could do reliably, although this was always precarious because of the effects on other areas of life.

*I’m great at working, but I get stuck on other things. And those things, other things that I’m stuck on eventually become things that affect me, like letting my health decline a little bit. Eventually means I suffer more from stress at work. – Joel.*

This difficulty did not only affect things that were aversive or difficult; it also included *“things I want to do and enjoy doing”* (Harriet). They mentioned struggling to start enjoyable work as well as leisure activities such as reading, gardening, exercise, and art:

*I’m keen on gardening as some people know but I think since the garden is an allotment it’s some distance away that involves me making all sorts of preparations to go out of the door and get there and so I do have some problems in initiating, getting, well, I have problems initiating deciding when I want to go but then I have problems with initiating and getting everything sorted out before I go. – Thomas.*

#### Difficulty Maintaining Relationships

Impact on ability to initiate interaction and maintain relationships was substantial. For some, this was the main problem caused by their inertia. One participant related their problems with initiating communication, even when it was not anxiety-provoking.

*I find keeping in contact with people really difficult. I know I should message a lot of people see if they are ok but can’t seem to initiate that first message. – Brian.*

Erin was one of several participants who related that, *“All relationships, all friendships in my life only work if the other person is prepared to do a massively disproportionate amount of the initiating, almost all of it.”* Some relied on routines, as described in Section “Lack of Voluntary Control,” to maintain relationships:

*I can really only do a friendship where the other person is willing to commit to seeing me on a regular schedule, like we’ll always see this day or whatever […] And even if it’s just well they’ve gone, ‘well let’s just agree that we will try and see each other once a month.’ Well they can’t do that. I can do once a week and I can do once a week and maybe some weeks we try but we can’t do it, but I can’t do [once a month]. – Alex.*

Relationships could also be strained by others’ difficulty understanding why the autistic person was not getting more done. The judgment of others could make the difference between acceptance and a negative experience:

*I am not sure it’s ALWAYS negative… Like sometimes I just embrace it and go with it and accept that that’s a month where I’ll be in one corner of the sofa, eating junk food and playing video games, because that’s all I can manage. It becomes negative when I try to force myself out of it or put it under the scrutiny of external judgment from other people. – Margaret.*

## Discussion

This study is a broad preliminary investigation into the experience and impact of autistic inertia. It arises from the concerns of autistic people, including the lead author, some of whom have said that this is the most disabling aspect of their autism (Murray, 2017). This study is unique in considering difficulty with tasks of any type, not exclusively social, and by specifically looking at difficulty acting on intentions, rather than sensory or motor experiences more broadly. Furthermore, this study gathers focused qualitative data directly from autistic people who are able to describe their own experiences. From those descriptions, we have found that difficulty acting on intentions arises from associated tendencies to resist stopping, starting and changing activity. While difficulty with planning and prioritizing was common, a subset of participants described a more profound impairment in initiating even simple actions. Participants described complex interactions between various external and internal factors and their ability to act. What was consistent and universal among our participants was that the inability to start and stop activities at will had profound and pervasive effects on their day-to-day lives and general wellbeing.

### Characteristics of Initiation Impairments

The first goal of this research was to document the difficulties that autistic people experience acting on their intentions, which will both help in understanding these impairments and point out possible avenues for further research. The characteristics of these initiation impairments will be considered in terms of emotion and motivation, executive function, and movement.

#### Emotion and Motivation

While autism is now recognized as a neurological condition, there is still a tendency to view autistic behavior as social, emotional and volitional rather than the manifestation of a differently functioning brain. Too often, autistic people are considered non-compliant or unmotivated when they fail to act. It would be easy to attribute their inaction to laziness or lack of motivation; however, several characteristics of autistic inertia distinguish it from voluntary task avoidance. First, while one may procrastinate about doing a chore that is aversive, inertia also affects activities the person enjoys. Second, even for tasks that are difficult or unpleasant, a strong enough motivator can activate an avoidant person. By contrast, participants in our study could not overcome their inertia in order to carry out a task that was important to them, often even those driven by basic needs. Third, our participants experienced as much difficulty stopping as starting, so they were not simply avoiding effort. And finally, rather than enjoying their diversion from an undesirable activity, our participants were often frustrated, annoyed and even physically uncomfortable due to their inability to act. While transient lack of motivation and avoidance of undesirable tasks is a normal part of life, this debilitating level of initiation impairment affecting even simple and enjoyable actions is clearly beyond the typical experience.

There are several possible explanations for these experiences, aside from avoidance or non-compliance. For example, negative emotions and inaction were connected in a self-perpetuating cycle, where failing to do things created bad feelings which, in turn, made it more difficult to act. These factors are not unique to autism, but depression and anxiety occur at high rates in autistic adults (Hollocks et al., 2010; Hudson et al., 2018) including our participants. Nevertheless, our findings highlighted that initiation impairments cannot be entirely explained by motivational or emotional factors. Where anxiety did feature, it was not always clear whether it was causal; sometimes it seemed as if the person assumed anxiety was the cause because they could find no better explanation for failing to act.

For our participants, the most profound episodes of being ‘stuck’ were also the least likely to be connected with strong emotion. Catatonia-like physical freezing was often accompanied by blunted or absent thoughts and emotions. Although stress and anxiety could make episodes more likely, the overwhelming anxiety or depression reported by others (Paterson, 2016), such as being ‘frozen with fear’ or deeply unmotivated due to low mood, were not a proximal feature of these episodes, which were more often characterized by emotional detachment. Their lack of emotional arousal was remarkable given that they were often conscious of mounting discomfort (e.g., thirst and pain) and unpleasant consequences of failing to act. During such episodes, our participants also often experienced altered awareness of self, the environment and the passage of time. This was distinct from being absorbed in an activity where they may ‘lose track of time’ in that the person had reduced or absent ability to initiate voluntary movements. They often felt disconnected from their body and actions in a way that resembled dissociative experiences (Ben Shalom, 2000). Dissociation is associated with stress and trauma, an area of increasing interest in autism research (Brenner et al., 2018), and more than one third of our sample reported a current or past diagnosis of Post-Traumatic Stress Disorder. Further investigation is indicated to clarify any relationship between dissociation and the detachment experiences described by our participants.

Participants described with poignant clarity the profound impact of these difficulties, which have so far escaped the notice of most autism researchers and clinicians. More fundamentally, the incomprehensibility of *why* they didn’t ‘just do things,’ affected their self-concept as capable people. As autistic blogger, Sparrow (2016), writes, “it’s hard not to feel lazy or inadequate about one’s own inertia without the proper understanding of what it really is and what it really means.” The ability to respond to one’s environment at will is intimately connected with social interaction, agency, and identity.

#### Executive Function

Rather than an emotional basis, it is possible that difficulty initiating can be an outcome of executive dysfunction. Executive function is a diffuse concept with highly varied profiles found in previous research with autistic people (Demetriou et al., 2018). Flexibly starting, stopping and switching tasks depends on executive function (Hoofs et al., 2018; Yeung and Chan, 2020). Some of our participants had difficulty breaking down a task, but more often they broke it into so many components that it became overwhelmingly complex and impossible to find the starting point. This tendency to excessively segment a task may be a manifestation of autistic detail orientation (Mottron et al., 2006). The ease with which participants could be derailed from an activity may suggest a weakness in working memory or high distractibility. Those who experienced problems with sequencing a task benefitted from help finding the first step, which is consistent with experimental research finding an initiation-specific executive function impairment that could be overcome by providing the first step (Carmo et al., 2017). Prior research has investigated executive functioning deficits in autism, but our research is unique in considering this from an autistic perspective in an ecological context, which highlights the profound impact on accomplishing tasks in everyday life.

Difficulty switching between actions can be problematic when stuck in an inactive state, but a strong fixed attentional focus, sometimes referred to as ‘monotropism’ (Murray et al., 2005), also facilitates highly productive periods and a deep immersion in nature and hobbies. This experience is similar to ‘flow states’, which autistic people may experience from atypical sources, such as when engaging with specialist interests (Milton, 2017). Our participants occasionally experienced paradoxical bursts of high levels of productivity. These periods were described both as enjoyable immersive flow states and as panic-driven hyper-productivity. Such focused immersion can become problematic when it is so intense that it overrides shifting attention to other necessary or desired tasks. Nonetheless, in itself, a narrow focus is a natural and non-pathological aspect of autism, and attempts to overcome inertia by teaching the autistic person to be more flexible or engage in more varied activities would be misguided. The difficulties of autistic inertia need to be supported so that the related positives can be fully appreciated. Further research is needed to clarify the nature of the relationship between autistic inertia, resistance to change, and intense focus.

#### Movement

Rather than being primarily a cognitive, emotional or social deficit, both the failure to act and the lack of response to that failure could at times be due to an impairment of voluntary motor initiation. This difficulty shares characteristics with those of autistic catatonia described by Shah (2019), as detailed in Table 3, but often more subtle. This type of initiation difficulty is manifest as a loss of conscious voluntary control of goal directed action affecting even simple, familiar actions such as standing up from a seated position or reaching for a drink. A further distinctive characteristic of this type of experience is the response to interruptions. When experiencing inertia characteristic of executive function impairments, interruptions were perceived as an annoyance and avoided if possible as participants found it difficult to return to the original task. Conversely, when in a disconnected catatonic state, a small interruption such as a noise from another person could trigger an end to the episode of immobility.

Due to the limited communication abilities of those affected, the existing literature on catatonia is entirely by carer report and observation. In one such study, Breen and Hare (2017) found difficulty initiating actions was the least common of six ‘core’ catatonia symptoms. However, as intentions are invisible, and a lack of emotional arousal and an intact ability to make other movements could mask the unrealised intention to move, difficulty with initiation may be underestimated by carer reports. For this reason, such phenomena can only be fully explored through the subjective experience of autistic individuals. Broader experiences of autistic embodiment, including motor and arousal control, have been explored through first-hand accounts by autistic people (Welch et al., 2018, 2020). In these accounts, autistic people also report a variety of difficulties with controlling their action and inaction, including feeling a ‘mind-body disconnect.’ By specifically asking autistic participants about such episodes, the present study provides unique insight into the internal experience, and the ability of our participants to fully articulate these experiences may be further enhanced by their connections to and interactions with the autistic community.

While there is some value in considering different approaches to initiation problems that have a primarily emotional, executive function, or movement profile, these are not completely dissociable. The association between anxiety and catatonia (Shah and Wing, 2006, p. 250) is inconsistent, with some studies reporting high levels of anxiety in up to 80% of catatonic patients (Northoff, 2002) and others reporting none (Pelzer et al., 2018). Furthermore, impairments in executive function, movement and motivation (variously called apathy, avolition or initiative impairment depending on the area of study) co-occur in a variety of neurological and psychiatric conditions including parkinsonism, depression and schizophrenia (Yamanaka et al., 1996; Ozonoff and Jensen, 1999; Bertilsson et al., 2018). Given these associations and the overlapping cortico-striatal circuitry involved in cognitive flexibility and movement control (Daniels, 2009; Uddin, 2021), these may be compatible rather than competing explanations. Teasing these apart and specifying the relationship between them is beyond the scope of this paper but should be explored in further research as they may lead to understanding of the mechanisms and interventions for the most debilitating of autistic initiation impairments.

### Implications for Understanding and Supporting Autistic People

Autism is currently characterized as a dyad of impairments in (i) social interaction, and (ii) flexibility (American Psychiatric Association, 2013). A prominent finding which may be surprising to those who view autism as primarily defined by social deficits was that difficulty maintaining relationships was one of the most frequently mentioned negative impacts of their initiation impairment. Contrary to the view that autistic people initiate interaction less often because they are less interested in others (Chevallier et al., 2012; Kohls et al., 2012), participants in our study wanted to contact people who were important to them, but found themselves unable to initiate. In respect to the other aspect of the core dyad, our research suggests that resistance to change relates not only to repetitive motor mannerisms and resistance to transitions imposed by others, but also with starting and stopping internally motivated actions.

The experiences described in the ‘disconnection between intention and action’ theme support the view of a small number of researchers who propose that many autistic characteristics may be attributed to sensorimotor differences (Robledo et al., 2012; Donnellan et al., 2013; Torres and Donnellan, 2015). Understanding the role of various factors underpinning difficulty initiating action can enable more successful support strategies. The core characteristics of inertia and answers to the focus group question ‘what helps’ have led to some principles to consider when trying to assist an autistic person struggling to initiate tasks, which are described in Table 4.

**TABLE 4:** Principles for helping with autistic inertia.

Principle	Explanation and examples
**Distinguish between mechanisms** Consider whether the current difficulty acting is underpinned by motivational/emotional, organizational or movement problems, because they have different responses to support.	• *Motivational:* tasks that are stressful, aversive, or anxiety inducing. • *Organizational:* tasks that are complex or involve transitions. • *Movement:* can affect even very simple tasks and meeting basic needs.
**Use continuity** When the autistic person wants to continue with a task, make it easy to continue.	• Avoid interruptions, e.g., provide all information necessary to make a decision at the time the question is asked. • Avoid unnecessary transitions and interruptions. • Keep moving, e.g., avoid sitting down between active tasks.
**Use prompts carefully** Prompting can be helpful, but if used incorrectly can exacerbate difficulties.	• Sensitively delivered without adding stress. • During natural breaks in attention. • To break away from disconnected passive states. • Avoid nagging to attend to others’ priorities as such demands are stressful and exacerbate issues.
**Environmental scaffolding** Provide an environment that supports action	• Do tasks in an environment specific to those activities, e.g., working in a designated study or office. • Engage in compatible activity nearby. • Keep a regular routine.
**Lower the threshold** Make it easier to start by lowering the initial hurdle	• Self-talk or encouragement to only do one small step in the desired direction. • Have someone else do the first step.

*Outline of five key principles to apply the results of this study to assisting autistic people to initiate tasks. Five key principles (bold) to apply the results of this study to assisting autistic people to initiate tasks. Each is accompanied by a brief description and examples of practical applications where appropriate.*

Participants almost universally found that conventional organization and memory tools such as alarms, lists, reminders and calendars were seldom helpful; practical assistance was far more beneficial. Initiation impairments were often related to the height of the cognitive threshold to overcome, so it was more difficult to get out of bed than to pick up a phone, and complicated activities such as leaving the house were especially difficult. Having another person provide all necessary information or start off the task lowered the initiation threshold, thereby facilitating action.

Social connections were not only one of the most significant casualties of their impairments, but also very important in mitigating the effects of initiation impairments. Prompting from another person in their presence was the most helpful intervention. Even having someone working nearby without interacting was often helpful. This is consistent with evidence from executive function research (Williams et al., 2014) and similar effects have been reported in books on catatonia by Sacks (1976) and Shah (2019, p. 108). Participants also found it easier to do anything where another person was depending or counting on them, even from a distance, and most difficult to do something only for themselves. Mistimed or misdirected prompts or excessive demands and pressure could cause stress which would exacerbate issues. This also applied to internally generated pressure such as self-imposed ‘deadlines’ and schedules. Several participants had developed personal techniques to reduce the pressure of expectation. For example, by telling themselves *“all you have to do is…”* one tiny step, they could circumvent the sense of pressure and demands that could cause them to get stuck.

### Limitations and Next Steps

While providing novel insights, this study is limited by the inherent limitations of a broad research question. This has not allowed for detailed inquiry about the influence of gender, co-occurring diagnoses, personal history, or other differences. We hope that this research will inspire others to look at autistic inertia further, including these nuances.

The participants in this study should not be considered representative of the autistic population as a whole. The focus on internal experiences excludes those who are unable to reflect on or express their experiences in an accessible way. Although Welch et al. (2018) found overlapping themes in the memoirs of minimally verbal autistic young people, the experiences of those who do not use the written or spoken word to communicate remain inaccessible to this form of enquiry. Those who attended the face-to-face groups are more likely to be sociable and to tolerate participation in groups. This was offset by also conducting text-based online focus groups which included people who were unable to attend Autscape or participate in a verbal group interaction. Our participants may also have been more likely to attend if they experienced difficulty with inertia, as the purpose of this study was to describe the phenomenon rather than to draw inferences about prevalence.

Recruitment of a majority of participants from the Autscape event also limits the range of the sample. Autscape participants are more likely to be introspective about their experiences of autism and to have communicated about them with other autistic people. While this limits the representativeness, it is also an advantage for this early enquiry. One of the goals of this study is to provide language to express experiences of being unable to act. By drawing from a community where autistic people share and develop their understanding of autism, they are more likely to have developed ways of reflecting on and expressing their experiences and strategies to overcome difficulties. The text-based focus groups included participants who had never attended Autscape, yet the themes were very similar, with no novel themes arising in these groups.

Possible future directions include exploring experiences of autistic inertia in the context of gender, living circumstances, support needs and co-occurring diagnoses, which were not considered in the current study. Although there has been some quantitative research characterizing executive deficits in autistic people, further research is needed to understand the impact of these on day-to-day life, including aspects of inertia such as those described by the ‘difficulty finding the first step’ and ‘tendency to maintain one state’ themes and possible overlap with ADHD traits. Furthermore, the present study has indicated directions for investigation into possible associations with stress, dissociation, avolition, catatonia, and the possible underlying cortico-striatal circuitry. An increased understanding of these may help to tease apart the different mechanisms, improve understanding of these issues, and begin to work toward helpful interventions.

The lead researcher’s personal experience of severe initiation impairments suggestive of a movement disorder, and her personal interest and prior informal investigation of the topic may have colored interpretation of the data. However, this author’s personal interest and experience has also been an asset in understanding the issues and building rapport with participants. Future research should continue to adopt a participatory research framework.

This research was prompted by members of the autistic community who experience the disabling aspects of inertia. The absence of documented evidence that difficulty initiating action is part of the autistic experience hampers access to understanding and effective support. While the focus of interventions for autistic people is on anxiety and social issues, many supporters are not even aware that inertia can be ‘the single biggest problem’ (Murray, 2017) arising from autism, creating a life that is *“a lot smaller and less than”* (Sam, focus group) it should be.

## Data Availability Statement

The raw data supporting the conclusions of this article will be made available by the authors, without undue reservation.

## Ethics Statement

The studies involving human participants were reviewed and approved by University of Manchester Research Ethics Committee. The patients/participants provided their written informed consent to participate in this study.

## Author Contributions

KB conceived the idea and designed the investigation with support from EG, KL, and EP, who supervised the project. KB collected and analyzed the data and drafted the manuscript. All authors discussed the results and provided critical feedback which helped shape the research and analysis. All authors contributed to the final manuscript.

## Conflict of Interest

The authors declare that the research was conducted in the absence of any commercial or financial relationships that could be construed as a potential conflict of interest.

## Footnotes

1 This paper uses the term “autistic people” to reflect the preferences of a majority of autistic adults (Kenny et al., 2016), although the authors acknowledge that this preference is not universal, with some finding it offensive (Bury et al., 2020) and that research on the topic remains scarce (Shakes and Cashin, 2019).

2 In recognition of the fact that alternative gender identity and expression is common among autistic people (George and Stokes, 2018), the questionnaire asked all participants to self-describe their gender rather than providing specific options. Those who do not identify as either “male” or “female” often refer to themselves as “non-binary”. At times this paper will use the singular “they” to reflect the preference of some participants to avoid using gender-specific pronouns.

3 ‘Spoons’ here refers to ‘spoon theory’, a well-known analogy for fatigue and limited resources (Miserandino, 2003).
